# *Pseudomonas* and *Pseudarthrobacter* are the key players in synergistic phenanthrene biodegradation at low temperatures

**DOI:** 10.1038/s41598-024-62829-y

**Published:** 2024-05-25

**Authors:** Kallayanee Naloka, Aunchisa Kuntaveesuk, Chanokporn Muangchinda, Suchana Chavanich, Voranop Viyakarn, Bo Chen, Onruthai Pinyakong

**Affiliations:** 1 Center of Excellence in Microbial Technology for Marine Pollution Treatment (MiTMaPT), Department of Microbiology, Faculty of Science, Chulalongkorn University, Bangkok, 10330 Thailand; 2 Research Program on Remediation Technologies for Petroleum Contamination, Center of Excellence on Hazardous Substance Management (HSM), Bangkok, 10330 Thailand; 3 International Postgraduate Programs in Hazardous Substance and Environmental Management, Graduate School, Chulalongkorn University, Bangkok, 10330 Thailand; 4 Reef Biology Research Group, Department of Marine Science, Faculty of Science, Chulalongkorn University, Bangkok, 10330 Thailand; 5 Aquatic Resources Research Institute, Chulalongkorn University, Bangkok, 10330 Thailand; 6 Polar Biological Science Division, Polar Research Institute of China, Shanghai, China

**Keywords:** Antarctica, Bacterial community, Bioremediation, PAHs, Synergistic interactions, Biotechnology, Microbiology, Environmental sciences

## Abstract

Hydrocarbon contamination, including contamination with polycyclic aromatic hydrocarbons (PAHs), is a major concern in Antarctica due to the toxicity, recalcitrance and persistence of these compounds. Under the Antarctic Treaty, nonindigenous species are not permitted for use in bioremediation at polluted sites in the Antarctic region. In this study, three bacterial consortia (C13, C15, and C23) were isolated from Antarctic soils for phenanthrene degradation. All isolated bacterial consortia demonstrated phenanthrene degradation percentages ranging from 45 to 85% for 50 mg/L phenanthrene at 15 ℃ within 5 days. Furthermore, consortium C13 exhibited efficient phenanthrene degradation potential across a wide range of environmental conditions, including different temperature (4–30 ℃) and water availability (without polyethylene glycol (PEG) 6000 or 30% PEG 6000 (w/v)) conditions. Sequencing analysis of 16S rRNA genes revealed that *Pseudomonas* and *Pseudarthrobacter* were the dominant genera in the phenanthrene-degrading consortia. Moreover, six cultivable strains were isolated from these consortia, comprising four strains of *Pseudomonas*, one strain of *Pseudarthrobacter*, and one strain of *Paeniglutamicibacter*. These isolated strains exhibited the ability to degrade 50 mg/L phenanthrene, with degradation percentages ranging from 4 to 22% at 15 ℃ within 15 days. Additionally, the constructed consortia containing *Pseudomonas* spp. and *Pseudarthrobacter* sp. exhibited more effective phenanthrene degradation (43–52%) than did the individual strains. These results provide evidence that *Pseudomonas* and *Pseudarthrobacter* can be potential candidates for synergistic phenanthrene degradation at low temperatures. Overall, our study offers valuable information for the bioremediation of PAH contamination in Antarctic environments.

## Introduction

Although Antarctica is one of the most pristine areas on Earth, anthropogenic activities such as tourism and the establishment of scientific research stations have led to contamination with persistent organic pollutants (POPs) in the region^1^. Polycyclic aromatic hydrocarbons (PAHs) are a class of POPs that have raised concerns at the global level because of their toxic, mutagenic, teratogenic, and carcinogenic effects on organisms^2,3^. PAHs are distributed in all parts of the Antarctic environment, including the atmosphere^4^, water^1^, snow^5^, soil^6^, and sediment^7^. The main sources of PAHs in Antarctica are biomass and coal combustion, with PAHs primarily associated with the activities of scientific stations or transported to the region through atmospheric processes^1^. Han et al.^8^ determined the average concentrations of 16 PAHs in environmental samples from Ardley Island, Antarctica. They found that phenanthrene accounted for a high proportion, with concentrations ranging from 1.95 to 42.18 ng/g, representing 24–50% of the total PAHs. The concentrations of PAHs in soil from the vicinity of the Bulgarian Antarctic Station (St. Kliment Ohridski) were studied by Abakumov et al.^9^. In these works, the concentration of PAHs in soils ranged from 170 to 200 μg/kg. Therefore, remediation efforts are essential to mitigate the long-term impact of PAH contamination.

Biodegradation of PAHs in the Antarctic region by native microorganisms is considered to be a cost-effective and sustainable approach for PAH contamination remediation^10^. Moreover, the introduction of nonindigenous microbes is not permitted in the Antarctic region^11^. Therefore, the identification of indigenous Antarctic microorganisms with PAH-degrading capabilities is essential for the bioremediation of contaminated Antarctic environments. To date, various PAH-degrading bacteria have been isolated from Antarctica, including *Pseudomonas*, *Rhodococcus*, *Sphingobium*, and *Acinetobacter*^12,13^, which are common genera found in soils from both polar and temperate regions. Dietz-Vargas et al.^12^ isolated *Acinetobacter* sp. OHIG3-2 from soil collected near the Chilean Antarctic Station and found that it could degrade 18% phenanthrene at 28 °C within 4 days. In addition, many studies have indicated that the application of Antarctic bacterial consortia is efficient for bioremediation. To our knowledge, published studies have been focused mainly on the biodegradation of petroleum hydrocarbons by Antarctic microbial consortia^14,15^. Although recent studies have been focused on microbial community dynamics and researchers have attempted to identify the key bacterial players involved in the bioremediation process through high-throughput sequencing, there are still research gaps regarding the role of isolated strains in supporting this prediction. Sulbaran-Bracho et al.^6^ isolated the diesel-degrading bacterial consortia LR-30 and LR-10 from Antarctic rhizosphere soil. They found that the dominant bacterial genera of the LR-30 community were *Achromobacter*, *Pseudomonas* and *Rhodanobacter*, whereas those of the LR-10 community were *Pseudomonas, Candidimonas* and *Renibacterium*. van Dorst et al.^16^ investigated the microbial dynamics associated with large-scale bioremediation of hydrocarbon-contaminated soil in Antarctica and reported that the genera *Alkanindiges, Arthrobacter, Dietzia*, and *Rhodococcus* were responsible for hydrocarbon degradation.

Antarctic environments exhibit extreme climatic conditions characterized by low temperatures and low water availability, and these factors can inhibit or reduce metabolic activity in microorganisms, leading to low contaminant degradation rates. Furthermore, this region is affected by global warming, as evidenced by records showing a rise in surface water temperatures of approximately 1 °C in the Antarctic Peninsula between 1955 and 2004 and up to 2.3 °C over a century. A new all-time temperature record of 18.3 °C over the continental Antarctic region was observed on 6 February 2022^17^. Therefore, it is essential to have an in-depth understanding of microbial community structures and degradation potential or activity under various environmental conditions. Bacteria adapted to a broader temperature range may offer significant advantages in biotechnological applications. They have high scientific relevance, particularly for environmental monitoring and safeguarding these extreme ecosystems from anthropogenic impacts.

In this study, we attempted to enrich PAH-degrading bacterial consortia from Antarctic soils and examined their degradation potential under different environmental conditions. This is crucial because it provides insights into the adaptability and effectiveness of these consortia in degrading PAHs under various scenarios, including under various ranges of temperature and levels of water availability. Additionally, high-throughput sequencing was employed to explore changes in the bacterial community during biodegradation and to identify the key and potential contributors to PAH degradation in the enriched bacterial consortia. PAH-degrading bacterial strains were potentially isolated and investigated for their ability to biodegrade PAHs, both as individual strains and as part of constructed consortia, to facilitate comprehensive research. This study can contribute to the development of efficient bioremediation strategies for PAH-contaminated Antarctic environments.

## Materials and methods

### Soil samples and enumeration of total heterotrophic and PAH-degrading **bacteria**

The surface soil samples used in this study were collected around the Great Wall Station on King George Island in Antarctica during the 30th Chinese Antarctic Research Expedition (CHINARE30) in January 2014. The soil samples were taken at a depth of 0–10 cm from 20 locations (Table 1), were stored at 4 °C for enumeration of total heterotrophic and PAH-degrading bacteria as well as enrichment of PAH-degrading bacterial consortia and were stored at − 20 °C for DNA isolation and sequencing analysis. The description of the sampling locations is summarized in Table 1 and in a previous report^18^. The numbers of total heterotrophic and PAH-degrading bacteria present in the soil samples were determined by the most probable number (MPN) method in 96-well microtiter plates, as described by Muangchinda et al.^19^. Total heterotrophic bacteria were counted in Luria–Bertani (LB) medium; low-molecular-weight (LMW) PAH-degrading bacteria were counted in mineral salt medium (MSM)^20^ supplemented with 250 mg/L phenanthrene; and high-molecular-weight (HMW) PAH-degrading bacteria were counted in MSM supplemented with 250 mg/L pyrene. The plates were incubated at 15 °C for 7 days (for total heterotrophic bacteria) or for 14 days (for PAH-degrading bacteria). Total heterotrophic bacteria were analyzed in positive wells by turbidity, while PAH-degrading bacteria were analyzed by respiration indicators. The numbers of total heterotrophic and PAH-degrading bacteria were retrieved from an MPN table.

**Table 1:** Site description and results of the enumeration of the number of total heterotrophic bacteria, as well as phenanthrene- and pyrene-degrading bacteria present in Antarctic soil samples.

Sample	Site description	Location	Number of total heterotrophic bacteria (MPN/g soil)	Number of phenanthrene-degrading bacteria (MPN/g soil)	Number of pyrene-degrading bacteria (MPN/g soil)
P1	Around Great Wall station: On the mountain-back from the station	S 62 13 11 W 58 57 33	3.6 ± 0.0 × 10^5^	1.4 ± 0.8 × 10^5^	7.4 ± 0.0 × 10^4^
P2	Around Great Wall station: Close to the oil tanks (red tank)	S 62 13 10 W 58 57 26	4.6 ± 0.0 × 10^6^	9.3 ± 0.0 × 10^5^	1.1 ± 0.3 × 10^5^
P4	Niujiao: Close to penguin rockery and seal	S 62 10 28 W 58 59 29	3.5 ± 0.0 × 10^5^	1.8 ± 0.8 × 10^5^	4.6 ± 2.3 × 10^4^
P5	Niujiao: Coastal line close to S4	S 62 10 29 W 58 59 56	5.5 ± 3.2 × 10^4^	n.d	n.d
P6	Niujiao: Close to penguin rockery	S 62 10 28 W 58 59 4	2.9 ± 0.5 × 10^5^	n.d	n.d
P7	Around Great Wall station: In front of the building	S 62 12 58 W 58 57 38	1.7 ± 0.5 × 10^5^	7.4 ± 6.6 × 10^4^	3.6 ± 0.0 × 10^4^
P8	Ardley Island: Close to penguin rockery	S 62 12 38 W 58 55 41	1.1 ± 0.0 × 10^5^	9.2 ± 0.0 × 10^4^	7.4 ± 0.0 × 10^4^
P9	Ardley Island: Close to penguin rockery	S 62 12 41 W 58 55 57	1.1 ± 0.0 × 10^5^	7.4 ± 0.0 × 10^4^	6.4 ± 4.0 × 10^4^
P10	Ardley Island: Close to penguin rockery	S 62 12 41 W 58 54 4	1.1 ± 0.0 × 10^5^	3.6 ± 0.0 × 10^4^	7.3 ± 3.2 × 10^4^
P11	Around Great Wall station: Near the road, on the beach	S 62 12 51 W 58 57 46	2.3 ± 0.0 × 10^5^	n.d	n.d
P12	Around Great Wall station: On the road, near Chinese character	S 62 12 42 W 58 57 45	2.1 ± 0.5 × 10^6^	1.4 ± 0.8 × 10^5^	7.4 ± 6.6 × 10^4^
P13	Around Great Wall station: On the road, near the station building	S 62 12 56 W 58 57 49	3.5 ± 0.0 × 10^5^	1.3 ± 0.6 × 10^5^	2.2 ± 1.8 × 10^5^
P15	Around Great Wall station: On the road, near moss	S 62 12 53 W 58 57 47	4.9 ± 3.8 × 10^5^	1.4 ± 1.0 × 10^5^	3.6 ± 0.0 × 10^4^
P16	Around Great Wall station: On the mountain-back from the station	S 62 13 14 W 58 57 27	1.1 ± 0.0 × 10^7^	6.1 ± 0.0 × 10^4^	4.0 ± 1.8 × 10^4^
P18	On the mountain: Near Great Wall Station	S 62 13 19 W 58 57 19	8.9 ± 3.7 × 10^6^	1.8 ± 0.8 × 10^5^	3.6 ± 0.0 × 10^4^
P19	On the mountain: Near Great Wall Station	S 62 13 25 W 58 57 58	3.5 ± 0.0 × 10^5^	n.d	n.d
P20	On the mountain: Near Great Wall Station	S 62 13 27 W 58 58 11	2.1 ± 0.0 × 10^5^	6.4 ± 4.0 × 10^4^	5.5 ± 2.7 × 10^4^
P21	On the mountain: Near Great Wall Station	S 62 13 32 W 58 57 42	3.1 ± 0.4 × 10^5^	n.d	n.d
P22	On the mountain: Near Great Wall Station	S 62 13 35 W 58 57 32	5.1 ± 1.8 × 10^4^	3.0 ± 0.0 × 10^4^	3.6 ± 0.0 × 10^4^
P23	Around Great Wall station: Around the construction area	S 62 13 5 W 58 57 36	3.5 ± 0.0 × 10^5^	7.3 ± 3.2 × 10^4^	6.1 ± 0.0 × 10^4^

n.d., the number of PAH-degrading bacteria could not be detected.

### Enrichment of PAH-degrading bacterial consortia from Antarctic soil

The enrichment of PAH-degrading bacterial consortia was performed following the methods of Sakdapetsiri et al.^20^. Briefly, a total of 10 g of each soil sample was added to 30-mL sterilized glass bottles supplemented with 100 mg/kg of a particular PAH (phenanthrene or pyrene) and incubated at 15 °C for 75 days. After the incubation period, 1 g of soil was taken from a glass bottle and added to 9 mL of MSM. Then, 100 µL of soil solution was spread onto MSM agar plates supplemented with 100 mg/L phenanthrene or pyrene and incubated at 15 °C. After two weeks of incubation, bacterial colonies were picked from the plates, added to 5 mL of MSM supplemented with 50 mg/L phenanthrene or pyrene and shaken at 200 rpm for 14 days at 15 °C. Additional tubes containing only PAH-supplemented MSM served as abiotic controls. After incubation, the culture broth, which appeared turbid and yellow/orange-colored compared with the control, was transferred to fresh medium containing PAH and cultured as described above. This step was repeated five times to obtain PAH-degrading consortia.

### PAH biodegradation experiment

The enriched bacterial consortia were cultivated in 45 mL of tenfold diluted LB and incubated at 15 °C for 2 days. Bacterial cells were centrifuged at 8000 rpm and 4 °C for 10 min, washed twice, and suspended in sterile 0.85% NaCl (w/v) solution. The turbidity of the cell suspension was adjusted to 1.0 for optical density at 600 nm, equivalent to an initial viable cell count of 10^6^ colony forming units per milliliter (CFU/mL). The cell suspension was shaken at 200 rpm and 15 °C for 24 h to allow the cells to utilize the accumulated nutrients before the PAH degradation experiment was initiated. The cell suspension (0.5 mL) of the enriched consortia was inoculated in a tube containing 4.5 mL of MSM supplemented with 50 mg/L phenanthrene. The initial cell concentration was 10^5^ CFU/mL. The tubes were incubated at 200 rpm and 15 °C. Uninoculated tubes containing MSM supplemented with 50 mg/L phenanthrene served as abiotic controls. Three test tubes were taken on days 0, 3 and 5 to analyze the residual phenanthrene content by performance liquid chromatography (HPLC) as described in a previous study^21^ and for DNA extraction as described in “DNA extraction and bacterial community structure analysis”. The HPLC system consisted of an LC-3A pump, an SPD-2A UV–visible detector and a C-RIA recorder (Shimadzu, Japan). Enriched bacterial consortia that exhibited the highest phenanthrene degradation efficiency were chosen for the PAH degradation tests. The tests involved assessing acenaphthene and fluorene (at 50 mg/L) degradation over 5 days and pyrene (20 mg/L) and benzo[*a*] pyrene (10 mg/L) degradation over 35 days. Uninoculated tubes containing MSM supplemented with PAHs served as abiotic controls. The cultures were incubated at 200 rpm and 15 °C. After cultivation, samples were collected for residual PAH analysis and DNA extraction.

### Effect of temperature and water content on phenanthrene degradation

An effective PAH-degrading consortium was selected for investigation of the effects of temperature and water content on phenanthrene degradation. Phenanthrene degradation was performed using the method described above. The bacterial cells were collected to study the bacterial community. The effect of temperature on phenanthrene degradation was determined at 4, 15 and 30 °C. The effect of water content on phenanthrene degradation was evaluated by withholding PEG 6000 or adding 30% (w/v) PEG 6000 to MSM. Samples were collected for residual phenanthrene analysis and DNA extraction. Uninoculated tubes containing MSM supplemented with phenanthrene served as abiotic controls.

### DNA extraction and bacterial community structure analysis

The genomic DNA of the enriched consortia was extracted in triplicate using a method described by Sharma et al.^22^. The extracted DNA was subjected to agarose gel electrophoresis and analysis via a NanoDrop™ 2000 spectrophotometer (Thermo Scientific, USA). The V3-V4 regions of the 16S rRNA gene were amplified using the primers 515F and 806R^23^. High-throughput 16S rRNA gene amplicon sequencing was performed according to previously described methods^24^. DNA libraries with dual indices were sequenced using an Illumina MiSeq platform (Illumina, CA, USA) with a 150 bp paired-end sequencing strategy. The raw sequences were processed and analyzed using QIIME 2 software tools version 2022.11 (https://library.qiime2.org). The reads were demultiplexed and qualified using the q2‐demux plugin and denoised with Deblur^25^. Taxonomic assignment was undertaken to obtain amplicon sequence variants (ASVs) using the q2‐feature‐classifier and classify‐sklearn naïve Bayes taxonomic classifier^26^ against the SILVA SSU taxonomic data operational taxonomic unit (OTU) reference sequences. The indices of alpha diversity, including the Shannon index, evenness, Faith's PD, and observed OTUs, were determined in QIIME2.

### Isolation of pure cultures from phenanthrene-degrading consortia

A tenfold serial dilution of the enriched consortia was prepared with 0.85% (w/v) NaCl solution. Then, the bacterial suspensions (10^−5^ to 10^−7^ dilution) were spread on LB plates and incubated at 15 °C for 3 days. Pure culture colonies were selected and then recultivated on LB agar. Colonies with different morphologies were selected, purified, and tested for their ability to degrade phenanthrene. All the isolated strains were subsequently identified via 16S rRNA gene sequence analysis. The genomic DNA was extracted and purified using the GenUP™ Bacterial gDNA Kit (Biotechrabbit GmbH, Germany) following the manufacturer’s instructions. The 16S rRNA gene was amplified using the universal 16S rRNA gene primers 27F (5ʹ-AGAGTTTGATCACTGGCTCAG-3ʹ) and 1492R (5ʹ-CGGCTTACCTTGTTACGACTT-3ʹ). The purified PCR products were sequenced by the Sanger method (First BASE Laboratories, Malaysia). The 16S rRNA genes of all the isolates were pairwise assigned to the reference sequences of the strains available in the EzBioCloud database (www.ezbiocloud.net/).

### Biodegradation of phenanthrene by the individual strains and constructed consortia

All the isolated strains were tested for phenanthrene degradation, and the results were compared between the individual strains and the constructed consortia composed of two isolated strains. The volume ratio of each strain was set to 1:1. The experiments were conducted in test tubes containing 4.5 mL of MSM supplemented with 50 mg/L phenanthrene. All the tubes were incubated at 15 °C and 200 rpm for 15 days. The phenanthrene remaining was extracted and analyzed via HPLC. Uninoculated tubes containing MSM supplemented with 50 mg/L phenanthrene served as abiotic controls.

### Statistical analysis

The residual phenanthrene concentration was expressed as the mean ± standard deviation of at least three replicates. One-way analysis of variance (ANOVA) and Duncan’s test were also conducted using SPSS software version 29 (https://www.ibm.com/spss) (SPSS, Inc., Chicago, IL, USA). *P* ≤ 0.05 was considered to indicate statistically significant differences.

## Results and discussion

### Quantity of total heterotrophic and PAH-degrading **bacteria** in Antarctic soil samples

The numbers of total heterotrophic and PAH-degrading bacteria present in the soil samples collected from twenty locations around the Great Wall Station are reported in Table 1. As shown in this table, the number of total heterotrophic bacteria in the soil ranged from 5.1 × 10^4^ to 1.1 × 10^7^ MPN/g soil. Among these samples, PAH-degrading bacteria were detected in 15 samples from 20 sampling sites. The number of phenanthrene-degrading bacteria ranged from 3.0 × 10^4^ to 9.3 × 10^5^ MPN/g soil, of which the greatest number was found in sample P2. The number of pyrene-degrading bacteria ranged from 3.6 × 10^4^ to 2.2 × 10^5^ MPN/g soil, and the greatest number was found in sample P13. Location P2 is close to the oil tanks, and location P13 is near the station building. These areas are active areas that may have been affected by human activities and contaminated with petroleum hydrocarbons. Pongpiachan et al.^18^ quantified the total concentrations of twelve PAHs, including phenanthrene, anthracene, fluoranthene, pyrene, benz[*a*]anthracene, chrysene, benzo[*b*]fluoranthene, benzo[*k*]fluoranthene, benzo[*a*]pyrene, indeno[1,2,3-cd]pyrene, dibenz[a,h]anthracene, and benzo[g,h,i]perylene, in soils collected around the Great Wall Station. Their results demonstrated that phenanthrene had the highest percentage contribution to these samples at 50%, followed by pyrene (18%) and fluoranthene (15.3%). Additionally, the total concentrations of PAHs varied from 0.296 to 10.4 ng/g. Hydrocarbon contaminants are known to induce an adaptive response in indigenous microbial communities. Therefore, it is of particular interest to isolate native bacteria capable of degrading hydrocarbons for bioremediating contaminated areas in the Antarctic region. This topic has become relevant due to the prohibition of bioaugmentation with foreign organisms in Antarctica. Furthermore, the presence of PAH-degrading bacteria in the environment can serve as an indicator of PAH contamination. *Sphingobium xenophagum* D43FB demonstrated effective phenanthrene degradation capability, achieving up to 95% degradation of 500 mg/L phenanthrene. This bacterium was also isolated from diesel fuel-contaminated Antarctic soils^13^.

### Enrichment of PAH-degrading consortia

**Figure 1:**
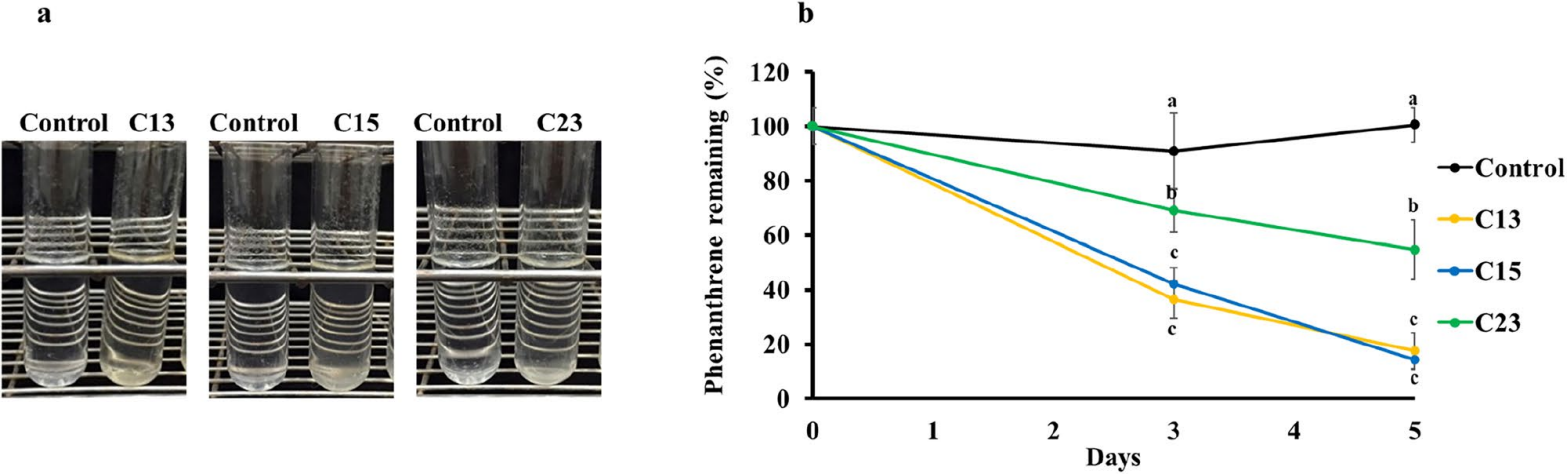
Color change in the phenanthrene-containing cultures after 14 days of incubation at 15 ℃ (**a**). Biodegradation of phenanthrene (50 mg/L) in liquid media at 15 ℃ by consortia C13, C15, and C23 over a period of 5 days. Different letters for the same day represent a significant difference at *P* ≤ 0.05 (**b**).

In this study, three phenanthrene-enriched bacterial consortia (C13, C15 and C23) exhibited changes in color in the culture compared to those of the control, indicating potential the biodegradation of phenanthrene (Fig. 1a). The ability of these consortia to degrade phenanthrene was tested, and the results of the biodegradation experiments are presented in Fig. 1b. Within 5 days, consortia C13 and C15 degraded 50 mg/L phenanthrene with efficiencies of 82.3% and 85.5%, respectively, at 15 °C, whereas the lowest phenanthrene biodegradation was recorded for consortium C23 (45.3%). Although microbial activity is generally inhibited or is lower at low temperatures^27^, these results demonstrate the capacity of cold-adapted bacteria to biodegrade phenanthrene under low-temperature conditions. However, no changes were observed in the color of the medium of the pyrene-enriched cultures. Sulbaran-Bracho et al.^6^ investigated the growth of consortium LR-10 on different PAHs at a concentration of 100 mg/L. LR-10 exhibited growth on anthracene and phenanthrene but not on pyrene after being incubated at 10 °C for 7 days. Pyrene is an HMW PAH, and biodegradation of this compound occurs more slowly than that of LMW PAHs such as phenanthrene^28^. Furthermore, the pyrene concentration in this study was greater than that in the Antarctic environment. Pongpiachan et al.^18^ reported that the average pyrene concentration in soils collected from the Great Wall Station was 0.570 ng/g. Although the PAH concentrations in Antarctic environments were previously reported to be low, recent studies have revealed an increased abundance of PAHs^9^. The concentrations of phenanthrene and pyrene used in this study are greater than those typically found in real Antarctic environments. Exposure to elevated PAH concentrations in laboratory settings may induce bacterial adaptation and evolution, resulting in the selection of specialized bacterial strains with enhanced biodegradation capabilities. In this study, consortia C13 and C15 exhibited effective degradation of phenanthrene, but the degradation efficacies of the two consortia were not significantly different. Therefore, these consortia were selected for the biodegradation of other PAHs, including acenaphthene, fluorene, pyrene, and benzo[*a*]pyrene.

### PAH biodegradation by enriched bacterial consortia

**Figure 2:**
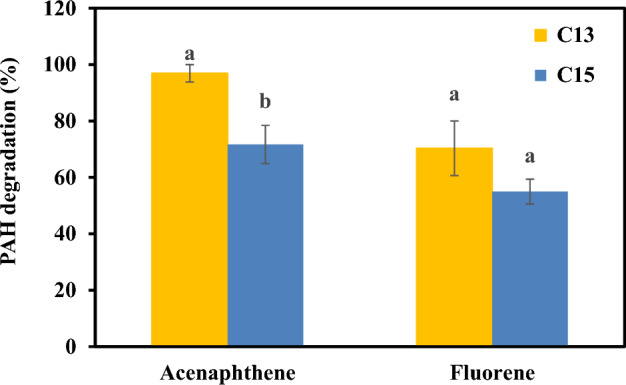
Biodegradation efficiency of 50 mg/L acenaphthene and fluorene in liquid media at 15 ℃ by consortia C13 and C15 after 5 days of incubation. Different letters for the same PAH represent a significant difference at *P* ≤ 0.05.

The degradation of four PAHs, LMW PAHs (acenaphthene and fluorene) and HMW PAHs (pyrene and benzo[*a*]pyrene), by consortia C13 and C15 was determined. Both consortia were able to degrade 50 mg/L acenaphthene and fluorene at 15 °C within 5 days (Fig. 2). Consortium C13 degraded 97% of the acenaphthene, which was significantly greater than the approximately 72% degradation achieved by consortium C15. The degradation percentages of fluorene by consortia C13 and C15 were 70% and 55%, respectively. However, these consortia could not degrade pyrene or benzo[*a*]pyrene. These results were consistent with previous studies in which HMW PAHs were more difficult to biodegrade than LMW PAHs^24,29^. Although few studies have reported the Antarctic bacterial consortia-driven biodegradation of PAHs other than phenanthrene, there is research on the biodegradation of the components of PAHs present in petroleum oil. Sulbaran-Bracho et al.^6^ reported the degradation of *n*-alkanes and PAH compounds in diesel oil by consortium LR-30 isolated from Antarctic rhizosphere soil. The consortium metabolized more than 90% of aliphatic compounds and 50% of naphthalene and pyrene after 7 days of incubation. Based on this evidence, we concluded that the obtained consortia consist of efficient degraders of LMW PAHs, which is consistent with the location from which they were isolated, i.e., from soils where LMW PAHs are abundant. In this study, consortium C13 effectively degraded various PAHs; therefore, this consortium was selected for further experiments.

### Effect of temperature and water availability on phenanthrene degradation

**Figure 3:**
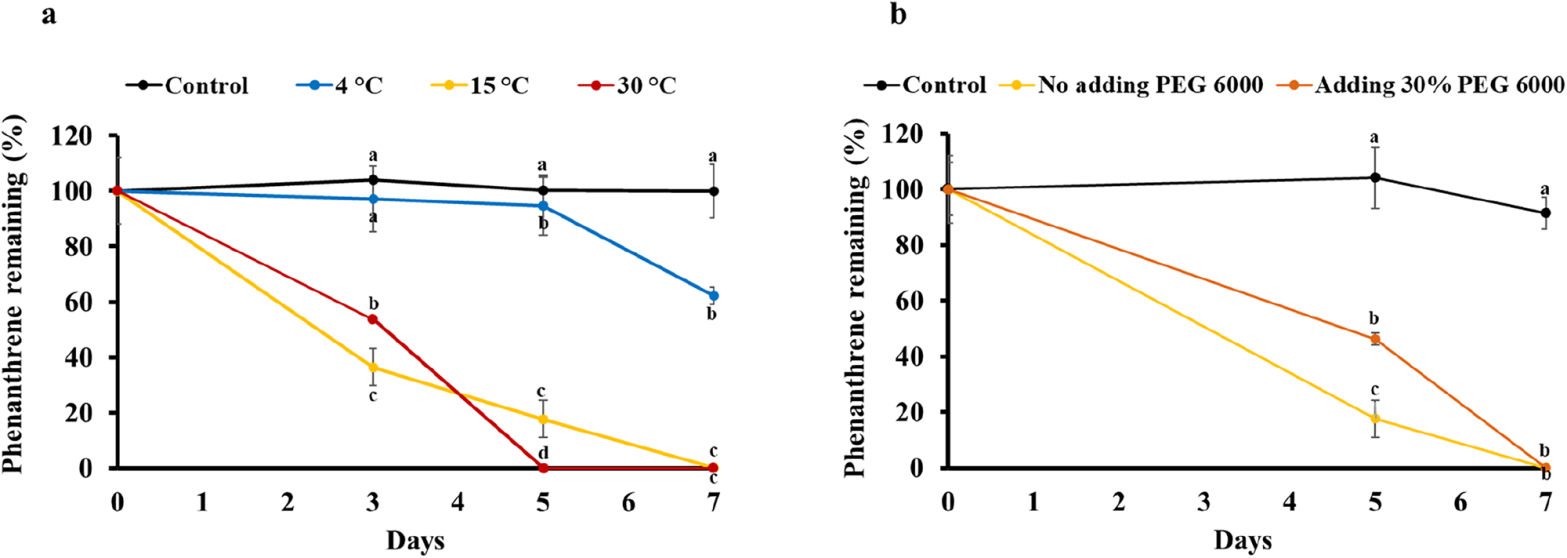
Biodegradation of phenanthrene (50 mg/L) by consortium C13 at different temperatures (**a**) and under different water availability conditions (**b**). Different letters for the same day represent a significant difference at *P* ≤ 0.05.

The biodegradation of PAHs is influenced by a variety of specific physical factors. Antarctic environments exhibit extreme climatic conditions characterized by low temperatures and low water availability^30^. Therefore, the effects of temperature and water availability on phenanthrene degradation by consortium C13 were evaluated. Temperature was shown to have an impact on biodegradation efficiency and the microbial community. Low temperature reduces biological activity and the rate of hydrocarbon degradation^31^. In this study, the greatest phenanthrene degradation was achieved at the highest incubation temperature (30 °C) (Fig. 3a). A decrease in temperature led to a delay or decrease in the degradation rate of phenanthrene. Consortium C13 could completely degrade 50 mg/L phenanthrene within 5 days at 30 °C and within 7 days at 15 °C. At 4 °C, 38% phenanthrene biodegradation was observed after 7 days of incubation. Vergeynst et al.^31^ studied hydrocarbon biodegradation at low temperatures and reported that the mineralization rates of hydrocarbons were 0.02%, 0.14% and 0.33% per day at 0, 4, and 15 °C, respectively.

To evaluate the effect of water availability on phenanthrene degradation, PEG 6000 was used to reduce the water content in the culture media. Poor water availability can limit PAH biodegradation because these conditions can limit the contact necessary between PAHs and microbes for biodegradation^32^. In this study, consortium C13 maintained high biodegradation efficiency under low water availability conditions (Fig. 3b); it completely degraded 50 mg/L phenanthrene at 15 °C within 7 days, both in the presence and absence of PEG 6000. Liu et al.^32^ determined the effects of water availability on the phenanthrene biodegradation rate and reported that the highest rate of mineralization was observed under the highest water content. Low-water-content conditions might limit nutrient diffusion and microbial movement, thus decreasing microbial activity and biodegradation. The ability of this consortium to maintain high biodegradation efficiency even under low water availability is noteworthy and contributes to our understanding of microbial degradation processes in challenging environments. To provide information on how bacterial consortia adapt to such extreme environments, we investigated their community responses.

### Bacterial community response during PAH degradation

**Figure 4:**
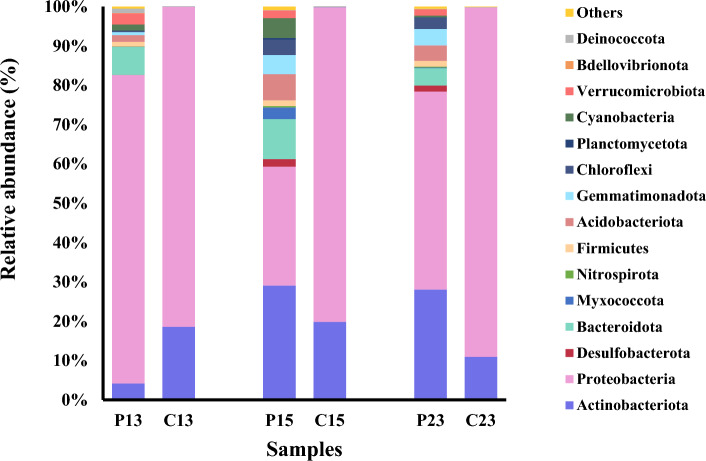
Bacterial compositions at the phylum level in the original soil and in the corresponding enriched consortia.

**Figure 5:**
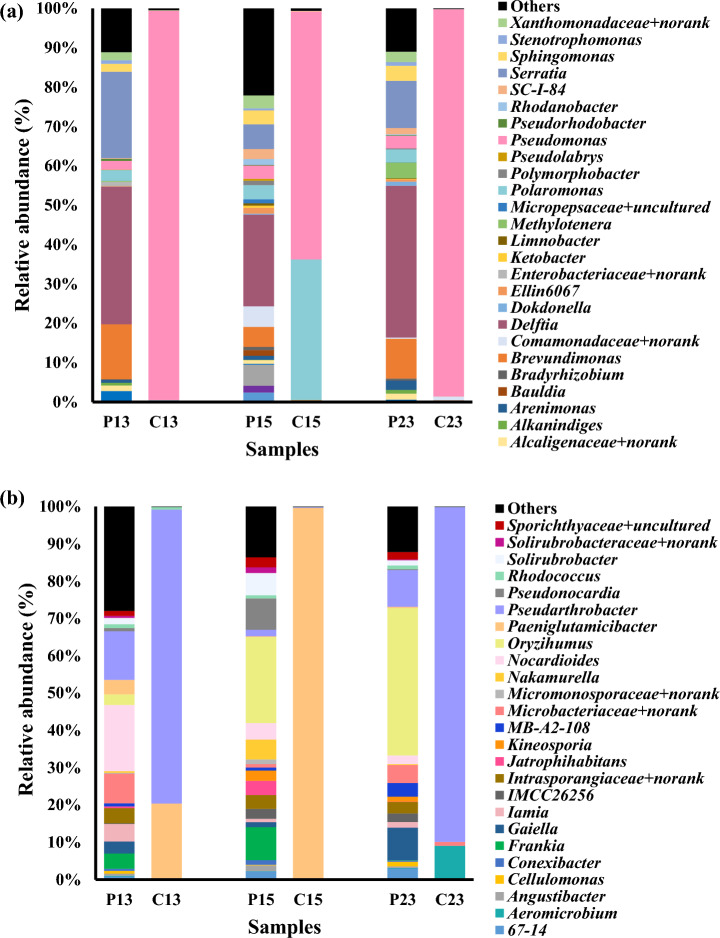
The relative abundances of bacteria belonging to the phyla Proteobacteria (**a**) and Actinobacteria (**b**) in the original soil and the corresponding enriched consortia at the genus level.

The bacterial communities in the enriched consortia and those in the corresponding original soil samples were characterized via high-throughput sequencing of 16S rRNA gene amplicons. The sequencing data can be accessed in the NCBI database (accession number: PRJNA1062590). The alpha diversity indices indicated lower bacterial diversity in the enriched consortia than in the original soil samples (Table 2). PAH contamination has been shown to influence and decrease the diversity and abundance of microbial communities^33^. The bacterial community structure of the original soil samples consisted of a total of 27 phyla, while the community in the enriched consortia consisted of a total of 6 phyla. Proteobacteria was the most abundant phylum found in the enriched consortia, comprising 80–89% of the total sequences in a sample. Actinobacteria was a minor phylum, accounting for 11–20% (Fig. 4). Proteobacteria and Actinobacteria have been reported to be the most abundant phyla in both Antarctic soils^34^ and soils contaminated with PAHs^35^. Figure 5a,b show all the genera belonging to the Proteobacteria and Actinobacteria phyla, respectively. After exposure to phenanthrene, the bacterial community structure of consortium C13 was dominated by *Pseudomonas* (Proteobacteria) (81%), *Pseudarthrobacter* (Actinobacteria) (15%), and *Paeniglutamicibacter* (Actinobacteria) (4%). In contrast, in consortium C15, *Pseudomonas* (50%), *Polaromonas* (Proteobacteria) (29%), and *Paeniglutamicibacter* (Actinobacteria) (20%) were dominant. On the other hand, the bacterial community structure of consortium C23 was dominated by *Pseudomonas* (Proteobacteria) (87%) and *Pseudarthrobacter* (Actinobacteria) (10%). The genera *Pseudomonas*, *Pseudarthrobacter*, and *Polaromonas* have been found in petroleum hydrocarbon-degrading communities isolated from cold environments. Li et al.^36^ reported that *Pseudomonas* exhibited the highest relative abundance in methylcyclohexane-degrading communities derived from Antarctic surface water. Sulbaran-Bracho et al.^6^ determined the community composition of bacteria during diesel biodegradation by consortium LR-10 isolated from Antarctic rhizosphere soil and found that the dominant bacterial genera were *Pseudomonas*, *Candidimonas*, *Rhodanobacter*, *Renibacterium*, *Pseudoarthrobacter*, and *Frateuria*. Jurelevicius et al.^35^ investigated the microbial communities present in hydrocarbon-contaminated soils from King George Island, Antarctica. They found positive correlations between the abundances of *Cytophaga*, *Methyloversatilis*, *Polaromonas*, and *Williamsia* and the concentrations of total petroleum hydrocarbons and/or PAHs.

**Table 2:** Diversity indices of the original soils and the corresponding enriched consortia.

Sample	Shannon	Evenness	Faith’s PD	Observed OTUs
P13: original soil	3.76	0.53	13.93	132
P15: original soil	6.53	0.86	18.43	198
P23: original soil	5.05	0.67	18.12	185
C13: enriched consortium	1.01	0.19	4.71	37
C15: enriched consortium	2.16	0.42	3.42	35
C23: enriched consortium	0.81	0.18	2.84	24

**Figure 6:**
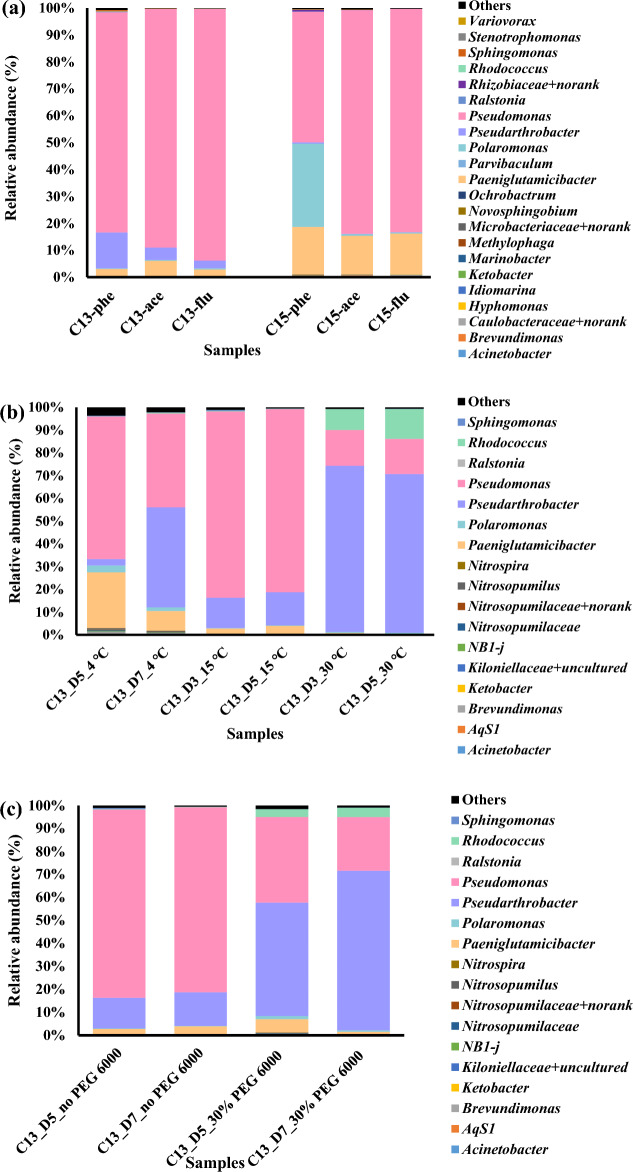
Bacterial compositions at the genus level under different substrate (**a**), temperature (**b**) and water availability (**c**) conditions.

The effects of different PAHs on the bacterial community structures were analyzed. The results showed that the community composition of consortium C13 did not change with exposure to different PAHs. However, the bacterial community structure of consortium C15 changed when exposed to different PAHs (Fig. 6a). The number of *Polaromonas* in the C15 community decreased when the consortium was exposed to acenaphthene and fluorene. PAHs can significantly impact the composition of bacterial communities, and some bacterial groups respond rapidly to changing environmental conditions. Ahmad et al.^37^ reported that PAH type significantly affects bacterial community composition and structure. The bacterial community compositions of the enriched consortia in the pyrene, benzo[*a*]pyrene, and benzo[*a*]fluoranthene treatments were significantly different from those in the phenanthrene treatments. This disparity might result from the greater toxicity of the former compounds compared to the latter or due to the inability of certain bacterial groups to utilize certain compounds as carbon and energy sources.

### Bacterial community composition of consortium C13 under different environmental conditions

Incubation temperature influenced the bacterial communities in consortium C13. At 15 °C, *Pseudomonas* had the highest relative abundance in the C13 microbial community, suggesting its potential role as the key phenanthrene degrader at this temperature (Fig. 6b). Several studies have revealed the ability of *Pseudomonas* to degrade phenanthrene. Ji et al.^38^ reported that *Pseudomonas* sp. Lphe-2 isolated from the aerobic sludge of a coking plant could degrade approximately 20% of phenanthrene (100 mg/L) at 15 ℃ within 7 days. However, when the temperature decreased to 4 ℃, the proportion of *Pseudomonas* also decreased. After 7 days of incubation, the abundance of *Pseudarthrobacter* increased, and this change was accompanied by the degradation of phenanthrene. In addition, when the temperature increased to 30 °C, the proportion of *Pseudarthrobacter* markedly increased, while that of *Pseudomonas* decreased. Moreover, the number of *Rhodococcus* bacteria increased under these conditions. Members of the genus *Pseudarthrobacter* have been shown to degrade phenanthrene. Li et al.^39^ reported that *Pseudarthrobacter* sp. L1SW was able to degrade 96.3% of 500 mg/L phenanthrene within 3 days at 30 °C. Moreover, studies have revealed that members of the genus *Pseudarthrobacter* can grow under a wide range of temperatures. For example, *Pseudarthrobacter albicanus* NJ-Z5^T^ isolated from Antarctic soil has been shown to grow at temperatures ranging from 4 to 28 °C^40^. Similarly, *Pseudarthrobacter humi* RMG13^T^ isolated from soil exhibited a capacity to grow within a temperature range of 4–37 °C^41^. Under poor water availability, the bacterial communities in consortium C13 were also dominated by *Pseudarthrobacter*, similar to the observations reported above (Fig. 6c). However, consortium C13 maintained high phenanthrene biodegradation efficiency at low water concentrations, indicating that *Pseudarthrobacter* might play an important role in phenanthrene degradation under conditions unsuitable for most other microorganisms. Muangchinda et al.^24^ investigated the impact of environmental conditions on the degradation of mixed PAHs by the SWO consortium. They found that the consortium retained its biodegradation capacity through alterations in the bacterial community structure and through adaptation to changing environmental conditions.

### Identification of pure strains isolated from phenanthrene-degrading consortia

Six cultivable strains were isolated from the phenanthrene-degrading consortia and taxonomically identified based on 16S rRNA gene sequence analysis. The sequences of all the isolates were deposited in the GenBank database under accession numbers OR889009–OR889014 (Table 3). Four of the six isolated strains belonged to the genus *Pseudomonas*. Strains ANT13_1 and ANT15_1 were identified as *P. silesiensis*, while strains ANT15_3 and ANT23_1 were identified as *P. frederiksbergensis* and *P. fildesensis*, respectively. Strain ANT13_2 was proposed as a representative novel species named *Paeniglutamicibacter terrestris*^20^. Strain ANT23_2 was closely related to the actinobacterium *Pseudarthrobacter humi*. These findings indicated that strains belonging to the genera *Pseudomonas* and *Pseudarthrobacter,* which were identified as the predominant genera in the enriched consortia based on the 16S rRNA gene amplicon sequencing results, were successfully isolated. Members of the genus *Pseudomonas* are the dominant PAH-degrading bacteria and are cold-adapted indigenous bacteria in Antarctic soils^42^. These isolated species were previously reported as cold-tolerant species^41,43–45^. Several species, including *P. silesiensis*^12^ and *P. frederiksbergensis* JAJ28^T^^45^, have been reported to be capable of growing and degrading phenanthrene. However, to our knowledge, phenanthrene degradation by *P. fildesensis*, *Paeniglutamicibacter terrestris* and *Pseudarthrobacter humi* at low temperatures has never been studied. To provide evidence for the potential involvement of the isolated strains in phenanthrene degradation, their degradation capabilities were investigated.

**Table 3:** Identification of phenanthrene-degrading isolates based on close similarity to the assigned species.

Strains	Accession number	Closest type strains (Accession no.)	Similarity (%)	Variation ratio
ANT13_1	OR889009	*Pseudomonas silesiensis* A3 (KX276592)	99.9	2/1401
ANT13_2	OR889010	*Paeniglutamicibacter terrestris* ANT13_2 (MT311166)	100.0	0/1445
ANT15_1	OR889011	*Pseudomonas silesiensis* A3 (KX276592)	99.6	6/1397
ANT15_3	OR889012	*Pseudomonas frederiksbergensis* JAJ28 (AJ249382)	99.6	6/1382
ANT23_1	OR889013	*Pseudomonas fildesensis* KG01 (MK859934)	99.7	4/1384
ANT23_2	OR889014	*Pseudarthrobacter humi* RMG13 (MZ031411)	99.3	9/1372

### Synergistic degradation of phenanthrene by the isolated strains

**Figure 7:**
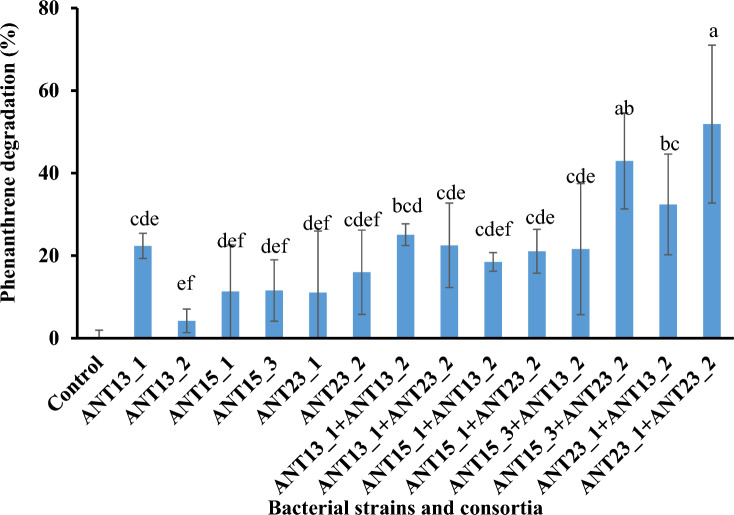
Biodegradation efficiency of 50 mg/L phenanthrene by individual strains and the constructed consortia after 15 days of incubation. The letters above the vertical bars represent significant differences (*P* ≤ 0.05).

Phenanthrene degradation (50 mg/L) during a 15-day incubation at 15 °C was compared among the individual strains and the constructed consortia, which consisted of bacteria in two genera, *Pseudomonas* and *Paeniglutamicibacter* or *Pseudarthrobacter* (Fig. 7). Among the individual strains, *Pseudomonas* sp*.* ANT13_1 exhibited the greatest phenanthrene degradation (22.4%). Similarly, for phenanthrene degradation by individual strains at low temperatures, *Pseudomonas* sp. JM2 degraded 12% of phenanthrene (50 mg/L) at 4 °C^46^. Previous studies have reported that *Pseudomonas* species possess PAH-degrading enzymes as well as cold-adaptive enzymes. For example, Song et al.^47^ reported that *P. fluorescens* S01 could degrade PAHs and heterocyclic PAHs under cold stress. The genome of this strain contains numerous systems for the catabolism of PAHs and heterocyclic PAHs and harbors numerous cold adaptation systems.

The constructed consortia significantly enhanced phenanthrene degradation, indicating that the strains had no inhibitory effects on one another. Two constructed consortia, ANT15_3 + ANT23_2 and ANT23_1 + ANT23_2, which consisted of *Pseudomonas* spp. and *Pseudarthrobacter* sp., exhibited high efficiencies of phenanthrene degradation, at 43% and 52%, respectively. The constructed consortium ANT23_2 + ANT13_2 (*Pseudomonas* sp. and *Paeniglutamicibacter* sp.) degraded 32.4% phenanthrene. The results indicated that the constructed consortia exhibited significantly greater phenanthrene degradation capabilities than the individual strains. In a previous study, *Kocuria flava* and *Rhodococcus pyridinivorans* were shown to degrade pyrene with efficiencies of 53.8% and 56.2%, respectively, within 15 days of incubation. Additionally, a consortium consisting of both strains achieved 56.4% pyrene degradation, indicating that the two strains did not inhibit each other^48^. Dechsakulwatana et al.^49^ reported that a constructed consortium consisting of S*phingobium naphthae* MO2-4 and *Bacillus aryabhattai* TL01-2 degraded approximately 43% of 50 mg/L phenanthrene within 7 days, while the individual strains degraded approximately 32–38%. Our results corresponded to the bacterial composition profile of the enriched consortia indicated that both *Pseudomonas* spp. and *Pseudarthrobacter* sp. are key degraders of phenanthrene at low temperatures (Figs. 5 and 6). A few reports have provided information on phenanthrene degradation by *Pseudarthrobacter*. Li et al.^39^ reported that *Pseudarthrobacter* sp. L1SW degraded 96.3% of 500 mg/L phenanthrene within 3 days at 30 °C. Additionally, there are reports indicating that *Pseudarthrobacter* species adapt to cold temperatures^50^. Therefore, this study serves as a starting point to show the synergistic ability of *Pseudomonas* and *Pseudarthrobacter* to increase phenanthrene degradation. These data are essential for developing potential bioremediation strategies to treat contaminated soil in cold areas for efficient pollutant removal. This is the first report on the use of a synthetic consortium of the genera *Pseudomonas* and *Pseudarthrobacter* isolated from Antarctic soil for effective phenanthrene degradation at low temperatures. A possible explanation for the synergistic effect is that when the two strains are cocultured, *Pseudomonas* spp. may increase the solubilization and enhance the bioavailability of phenanthrene by producing biosurfactants^51^. Furthermore, *Pseudomonas* spp. may provide protection against phenanthrene toxicity through biofilm formation and exopolysaccharide production. The Antarctic *Pseudomonas* sp. ID1 was reported to produce exopolysaccharides. These exopolysaccharides have a cryoprotective effect on *Pseudomonas* sp. ID1 and other bacterial cells^52^. Furthermore, *Pseudarthrobacter* species have been reported to possess specific survival strategies to cope with extreme environmental conditions, such as cold shock- and heat shock-protection genes^50,53^. Moreover, to enhance phenanthrene degradation by synthetic consortia, some minor bacterial genera, such as *Rhodococcus* and *Polaromonas*, identified based on bacterial community data of the 16S rRNA gene amplicon results, should be targeted in future isolation attempts from enriched consortia. A common challenge in previous studies was that some bacteria could not be cultivated by the methods used; moreover, slow-growing synergistic partners disappeared during isolation and/or because of the presence of metabolic dependencies in microbial communities^54,55^.

## Conclusions

Our findings demonstrated that Antarctic soils obtained from the Great Wall Station harbored bacteria capable of degrading PAHs at low temperatures. The enriched consortia derived from these soils exhibited high efficiency in phenanthrene biodegradation across a broad range of temperatures and at varying levels of water availability. Efficient phenanthrene degradation under cold conditions is noteworthy, particularly considering the challenges associated with bioremediation in polar regions. Furthermore, the environmental adaptability of the consortium enhances its potential applicability in diverse Antarctic habitats with varying environmental conditions. The results of 16S rRNA gene amplicon sequencing revealed that the phenanthrene-degrading consortia were dominated by *Pseudomonas* and *Pseudarthrobacter*. Both genera were successfully isolated from phenanthrene-degrading consortia, and these strains demonstrated the ability to degrade phenanthrene at low temperatures. Furthermore, constructed consortia, consisting of *Pseudomonas* spp. and *Pseudarthrobacter* sp., exhibited greater efficiency in terms of phenanthrene degradation than did the individual strains. These findings indicate that *Pseudomonas* and *Pseudarthrobacter* play important roles in phenanthrene degradation under low-temperature conditions. Additionally, these findings suggest that bacterial species can synergistically interact to enhance bioremediation efficiency, particularly in cold environments such as Antarctica. To gain a comprehensive understanding of the phenanthrene degradation pathway and the potential synergistic activity of these two strains, further studies must be conducted that include an evaluation of the intermediates of phenanthrene biodegradation and whole-genome analysis. Moreover, it is important to investigate the ability of these bacteria to produce biosurfactants, form biofilms in the presence of phenanthrene, and produce exopolysaccharides in addition to their cryoprotectant properties. Furthermore, to develop an efficient bioremediation strategy for Antarctic soils, bioaugmentation with the constructed consortia should be investigated to treat PAH-contaminated soils.

## Data Availability

The sequencing data of 16S rRNA gene amplicons presented in this study are available under NCBI BioProject ID PRJNA1062590. The bacterial 16S rRNA gene sequences are available under GenBank Accession numbers OR889009–OR889014.
